# Presepsin as a diagnostic biomarker for sepsis across neonates, children, and adults: A meta-analysis

**DOI:** 10.17305/bb.2025.12909

**Published:** 2025-08-20

**Authors:** Kaicheng Peng, Xiangmin Zhang, Qinyuan Li, Zhengxiu Luo

**Affiliations:** 1 Department of Respiratory Medicine, Children’s Hospital of Chongqing Medical University, National Clinical Research Center for Child Health and Disorders, Ministry of Education Key Laboratory of Child Development and Disorders, Key Laboratory of Children’s Vital Organ Development and Disease of Chongqing Health Commission, Chongqing Key Laboratory of Child Rare Diseases in Infection and Immunity, Chongqing, China; 2 Department of Radiology, Children’s Hospital of Chongqing Medical University, National Clinical Research Center for Child Health and Disorders, Ministry of Education Key Laboratory of Child Development and Disorders, Chongqing, China; 3 Chongqing Engineering Research Center of Stem Cell Therapy, Chongqing, China

**Keywords:** Biomarkers, presepsin, sepsis, meta-analysis, diagnosis

## Abstract

Sepsis remains a leading global health challenge, with delayed recognition and limited diagnostic accuracy of current tools contributing to high morbidity and mortality. Conventional clinical scores (SOFA/qSOFA), standard biomarkers (CRP, PCT), and blood cultures suffer from delayed responsiveness, insufficient specificity, or slow turnaround, underscoring the urgent need for more reliable early diagnostic strategies. Presepsin, a soluble CD14 subtype generated during pathogen recognition by innate immune cells, has emerged as a promising biomarker with potential to reflect infection status earlier and more specifically than traditional markers. This systematic review and meta-analysis quantitatively evaluated the diagnostic accuracy of presepsin across diverse populations. PubMed, EMBASE, Web of Science, and Cochrane Library were searched for studies published between 2015 and 2025. Forty-seven studies involving 7087 participants were included. Pooled sensitivity, specificity, diagnostic odds ratio (DOR), area under the curve (AUC), and likelihood ratios (PLR/NLR) with 95% confidence intervals (CI) were calculated using random-effects models. Heterogeneity was assessed with *I*^2^ statistics, meta-regression, and subgroup analyses. Study quality was evaluated using QUADAS-2. Presepsin demonstrated excellent overall diagnostic performance: pooled sensitivity 0.84 (95% CI: 0.81–0.88), specificity 0.86 (95% CI: 0.80–0.90), DOR 32.23 (95% CI: 20.11–51.66), and AUC 0.91 (95% CI: 0.88–0.93). Subgroup analyses confirmed robust performance across settings and populations, with particularly high accuracy in neonates (sensitivity 0.90, specificity 0.92, AUC 0.96), followed by children (sensitivity 0.84, specificity 0.81, AUC 0.88, NLR 0.20) and adults (sensitivity 0.81, specificity 0.82, AUC 0.87). Meta-regression identified year of publication, geographic region, specimen type, population, and diagnostic criteria as key contributors to heterogeneity, but sensitivity analyses confirmed result stability. No significant publication bias was observed (*P* ═ 0.33). In conclusion, presepsin is a valuable and highly promising biomarker for sepsis diagnosis, showing favorable diagnostic accuracy across populations, with strongest utility in neonates. Its application in pediatric and adult patients warrants further validation through large, prospective, multi-center studies.

## Introduction

Sepsis continues to pose a significant global healthcare challenge, impacting individuals across all age groups [1]. According to the World Health Organization, sepsis results in nearly 50 million cases and approximately 11 million deaths annually worldwide. This high mortality rate and substantial healthcare burden underscore the urgent need for more effective diagnostic and therapeutic strategies [2].

A major obstacle in clinical practice is the early identification and accurate diagnosis of sepsis. Tools such as the Sequential Organ Failure Assessment (SOFA) and quick SOFA (qSOFA) scores typically show abnormalities only after organ dysfunction has occurred, leading to significant diagnostic delays [3]. While traditional laboratory biomarkers, including C-reactive protein (CRP) and procalcitonin (PCT), are commonly employed, they are limited in their effectiveness. Their lack of specificity—elevated levels can occur in non-infectious systemic inflammation—and limited sensitivity, particularly in cases of localized infections or among immunocompromised patients, diminish their utility as early warning indicators [4].

Blood culture, regarded as the gold standard for pathogen identification, is time-consuming and often yields low positivity rates, failing to satisfy the urgent demand for rapid clinical decision-making [5]. Therefore, the discovery of novel biomarkers that can sensitively and specifically reflect the host’s immunopathological response during early infection is essential for facilitating timely detection and intervention, ultimately improving patient outcomes.

In this context, presepsin has emerged as a promising novel biomarker for diagnosing sepsis, garnering significant interest. The underlying biological mechanism involves pathogen invasion, during which surface components such as lipopolysaccharide bind to CD14 and toll-like receptor 4 (TLR4) on monocytes and macrophages. This binding event enzymatically cleaves CD14, leading to the release of presepsin into the bloodstream. In plasma, CD14 undergoes cleavage by cathepsin D, resulting in the formation of a smaller fragment known as presepsin. Plasma levels of presepsin increase following bacterial infections and decrease after antibiotic treatment [6]. Consequently, this molecule can be regarded as a marker of cellular immune response activation against pathogens. It indicates presepsin’s direct involvement in the earliest immune responses to pathogen recognition, suggesting it may reflect infection status earlier and more directly than secondary inflammatory products such as CRP or PCT [7, 8]. Presepsin secretion has also been linked to monocyte phagocytosis, implying that it could be measured in healthy, non-infected individuals and may reflect the activation of monocytes and macrophages in response to infections [9, 10]. Numerous studies have assessed the diagnostic performance of presepsin in sepsis patients, particularly within emergency departments and intensive care units (ICUs), often comparing it to traditional biomarkers [11].

However, existing research demonstrates significant heterogeneity in terms of sample size, detection methodologies, and patient populations. These variations have resulted in inconsistent findings regarding presepsin’s diagnostic efficacy. Therefore, this study aims to synthesize high-quality global clinical evidence to quantitatively assess the overall diagnostic accuracy of presepsin for sepsis and to investigate its performance across patient subgroups. This will provide robust evidence-based support for its potential role as an early clinical diagnostic tool to optimize sepsis management and improve patient survival.

## Materials and methods

### Search strategy

Retrieve English-language literature published between January 1, 2015, and May 31, 2025, focusing on the diagnostic value of presepsin for sepsis. A systematic literature search was conducted in the Embase, Web of Science, Cochrane Library, and PubMed databases using the combined terms: “sepsis,” “presepsin,” “sensitivity,” and “specificity.” The detailed search strategy is provided in the supporting information. Eligible citations from the retrieved articles were also screened. This study is registered with International Prospective Register of Systematic Reviews (PROSPERO), ID: CRD420251086778.

### Eligibility criteria

Studies were included based on the following criteria: (1) utilization of human-derived biological samples from patients diagnosed with sepsis and (2) provision of sufficient data to facilitate the direct or indirect calculation of true positive (TP), false positive (FP), false negative (FN), and true negative (TN) values. Exclusion criteria were as follows: (1) reviews, letters, meta-analyses, case reports, book chapters, or commentaries; (2) publications analyzing overlapping patient samples, indicating duplicate data reporting and (3) gray literature or preprints lacking peer review.

### Quality assessment

The Quality Assessment of Diagnostic Accuracy Studies-2 (QUADAS-2) tool [12] was utilized to evaluate methodological quality across four domains: 1) patient selection, 2) index test, 3) reference standard, and 4) flow and timing. Two authors (K.P. and X.Z.) independently rated each domain as high, low, or unclear risk. Any discrepancies in assessments were reconciled through consultation with a senior reviewer (Z.L.).

### Data extraction

Two investigators (K.P. and X.Z.) independently extracted the following parameters from the included studies: author, year, country, population, diagnostic criteria, study design, data sources, clinical setting, specimen type, analytical method, and sample size (case/control). Any discrepancies were resolved through consensus discussion with a third investigator (Q.L.), ensuring unanimous agreement on all extracted items. Diagnostic accuracy data (TPs, FPs, FNs, TNs) were obtained directly from 2×2 contingency tables or derived from reported sensitivity and specificity values. In studies that utilized both training and validation cohorts, only data from the validation set were extracted.

### Ethical statement

Ethical approval and written informed consent were not necessary for this study, in accordance with local and national guidelines.

### Statistical analysis

Statistical analyses were conducted using Stata 14 and RevMan 5.3. We calculated pooled sensitivity, specificity, positive likelihood ratio (PLR), negative likelihood ratio (NLR), and diagnostic odds ratio (DOR) along with 95% confidence intervals (CIs) [13, 14]. The DOR assesses the overall accuracy of a diagnostic test by comparing the odds of a positive result in diseased individuals to those in non-diseased individuals, using the formula: DOR ═ (TP × TN)/(FP × FN). The PLR reflects the extent to which a positive test result increases the likelihood of disease, defined as the ratio of sensitivity to (1 - specificity). Conversely, the NLR indicates the degree to which a negative test result decreases the likelihood of disease, calculated as: NLR ═ (1 - sensitivity)/specificity.

The diagnostic performance of presepsin was evaluated using summary receiver operating characteristic (SROC) curve analysis. Area under the curve (AUC) values were interpreted as follows: 0.7–0.9 indicates moderate accuracy, while values greater than 0.9 signify high accuracy. All metrics are presented as weighted proportions with 95% CIs. Heterogeneity was assessed using Cochran’s *Q* test and Higgins’ *I*^2^ statistic, with *I*^2^ > 50% indicating significant heterogeneity. Random-effects models were employed in cases of substantial heterogeneity; otherwise, fixed-effects models were used. Meta-regression and subgroup analyses were performed to investigate the effects of covariates on outcomes and sources of heterogeneity. Sensitivity analysis was conducted to evaluate the robustness of the study results. Publication bias was assessed using Deeks’ funnel plot asymmetry test. A two-sided *P* value of < 0.05 was considered statistically significant.

## Results

### Search strategy

**Figure 1.**
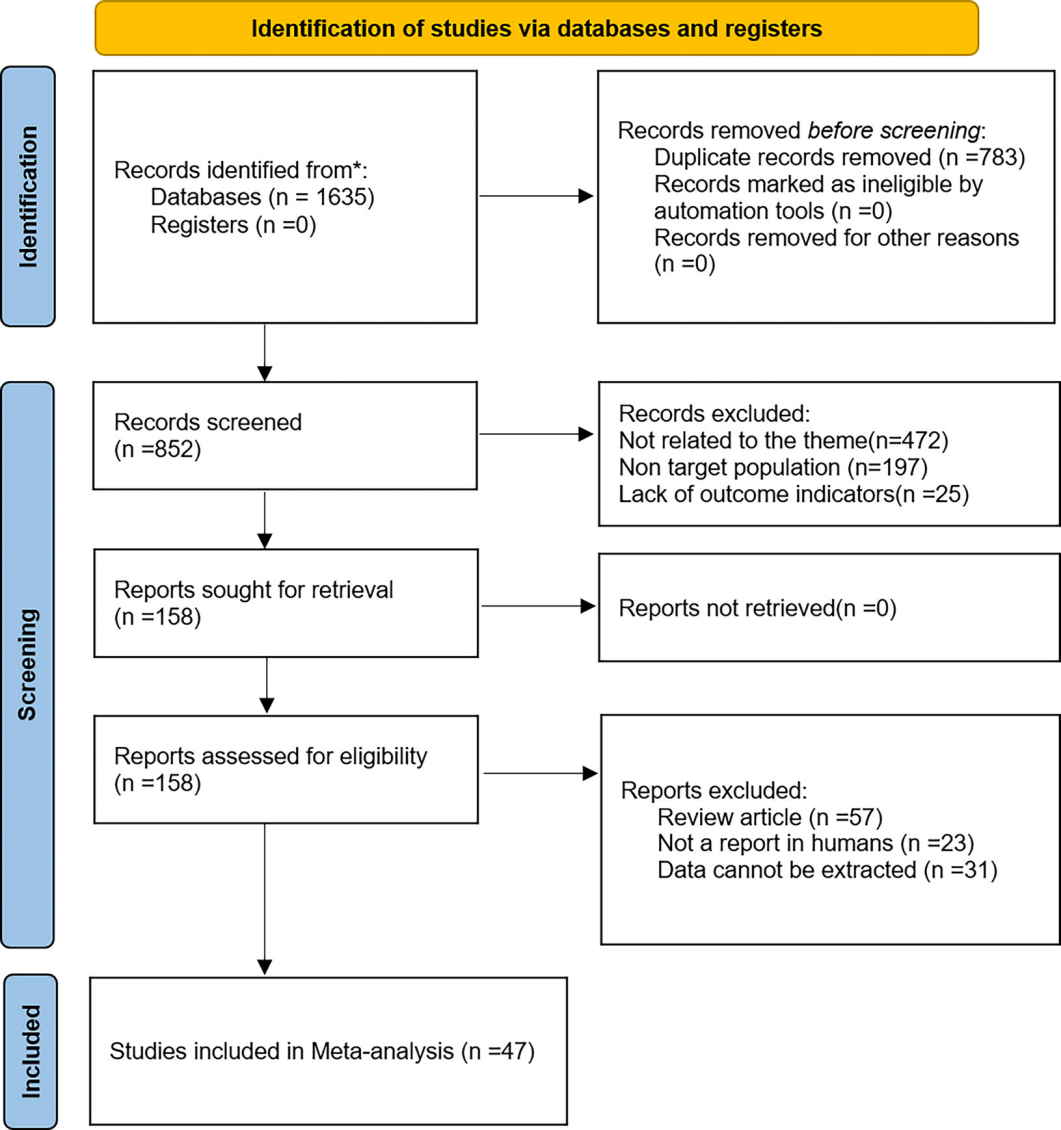
**Flowchart of literature search and studies selection**.

Our systematic search identified 1635 records. After removing 783 duplicates, 852 abstracts were screened. A total of 694 records were excluded: 472 were deemed irrelevant to the research topic, 197 were not reports on human subjects, and 25 lacked relevant outcome measures. A full-text assessment of 158 articles was conducted based on eligibility criteria. Exclusions included 57 review articles, 23 studies involving non-target populations, and 31 studies from which data could not be extracted, resulting in 47 qualifying studies (2015–2025) for inclusion [15–61]. Figure 1 illustrates the literature retrieval and selection workflow.

### Study characteristics

The analysis included 7087 participants across 47 studies (see Table 1). Temporal distribution revealed that 29 studies were conducted pre-2020, while 18 were conducted post-2020. Geographically, 22 studies originated from Asia and 25 from non-Asian regions. In terms of population distribution, the studies encompassed neonates (16 studies), children (5 studies), and adults (26 studies). Diagnostic criteria varied, with 19 studies utilizing positive blood culture, 6 employing Sepsis-2 criteria, 16 utilizing Sepsis-3 criteria, and 6 relying on clinical judgment. Methodologically, 44 studies were prospective, and 3 were retrospective. The majority of the studies (42 out of 47) were single-center, while 5 were multi-center. Clinical settings included ICUs in 37 studies and non-ICU settings in 10 studies. Specimen types included plasma (30 studies) and whole blood (17 studies). Analytical methods comprised chemiluminescent enzyme immunoassay (CLEIA) in 35 studies and enzyme-linked immunosorbent assay (ELISA) in 12 studies. Sample sizes varied, with 27 studies having fewer than 100 participants and 20 studies having 100 or more.

**Table 1:** Baseline characteristics of included studies

**Author**	**Year**	**Country**	**Population**	**Diagnostic criteria**	**Study design**	**Data sources**	**Clinical setting**	**Specimen type**	**Analytical method**	**Case/ Control**	**TP**	**FP**	**FN**	**TN**
Khater and Al-Hosiny [15]	2020	Egypt	Neonate	Positive blood culture	Prospective	Single-center	ICU	Plasma	ELISA	40/30	38	6	2	24
Xiao et al. [16]	2017	China	Neonate	Clinical judgment	Prospective	Single-center	ICU	Whole blood	CLEIA	42/53	40	8	2	45
Gad et al. [17]	2020	Egypt	Neonate	Clinical judgment	Prospective	Single-center	ICU	Plasma	ELISA	31/21	30	1	1	20
Ruangsomboon et al. [18]	2020	Thailand	Adult	Sepsis-3	Prospective	Single-center	Non-ICU	Plasma	CLEIA	139/111	116	42	23	69
Lu et al. [19]	2018	China	Adult	Sepsis-2	Prospective	Single-center	Non-ICU	Plasma	CLEIA	72/43	71	4	1	39
de Guadiana Romualdo et al. [20]	2017	Spain	Adult	Sepsis-3	Prospective	Single-center	Non-ICU	Plasma	CLEIA	130/70	87	13	43	57
Ali et al. [21]	2016	Egypt	Adult	Sepsis-3	Prospective	Single-center	ICU	Plasma	CLEIA	33/18	23	3	10	15
Sargentini et al. [22]	2015	Italy	Adult	Sepsis-2	Prospective	Single-center	ICU	Plasma	CLEIA	60/44	52	12	8	32
Takahashi et al. [23]	2015	Japan	Adult	Sepsis-2	Prospective	Multi-center	Non-ICU	Whole blood	CLEIA	359/489	285	82	74	407
Miyosawa et al. [24]	2018	Japan	Neonate	Positive blood culture	Prospective	Multi-center	ICU	Whole blood	CLEIA	13/18	11	2	2	16
Chen et al. [25]	2017	China	Neonate	Clinical judgment	Prospective	Single-center	Non-ICU	Whole blood	CLEIA	96/53	92	8	4	45
Montaldo et al. [26]	2017	Italy	Neonate	Positive blood culture	Prospective	Single-center	ICU	Whole blood	CLEIA	32/38	30	0	2	38
Ozdemir and Elgormus [27]	2017	Türkiye	Neonate	Positive blood culture	Prospective	Single-center	ICU	Whole blood	CLEIA	29/40	23	10	6	30
Mussap et al. [28]	2015	Italy	Neonate	Positive blood culture	Prospective	Single-center	ICU	Whole blood	CLEIA	25/25	25	5	0	20
Topcuoglu et al. [29]	2016	Türkiye	Neonate	Positive blood culture	Prospective	Multi-center	ICU	Whole blood	CLEIA	42/40	28	0	14	25
Iskandar et al. [30]	2019	Indonesia	Neonate	Positive blood culture	Prospective	Single-center	Non-ICU	Plasma	CLEIA	35/16	30	5	5	11
Kumar et al. [31]	2019	India	Neonate	Clinical judgment	Prospective	Single-center	ICU	Whole blood	ELISA	41/41	40	2	1	39
Rashwan et al. [32]	2019	Egypt	Neonate	Positive blood culture	Prospective	Single-center	ICU	Plasma	ELISA	98/66	84	0	18	66
Değirmencioğlu et al. [33]	2019	Türkiye	Neonate	Positive blood culture	Prospective	Single-center	ICU	Plasma	ELISA	26/29	23	3	3	26
Abdelshafey et al. [34]	2021	Egypt	Adult	Sepsis-3	Prospective	Single-center	ICU	Whole blood	CLEIA	26/14	19	1	7	13
Juneja et al. [35]	2023	India	Adult	Sepsis-3	Retrospective	Single-center	ICU	Whole blood	CLEIA	70/30	54	10	16	20
Poggi et al. [36]	2015	Italy	Neonate	Positive blood culture	Prospective	Single-center	ICU	Whole blood	CLEIA	19/2	18	0	1	2
Sakyi et al. [37]	2020	Ghana	Children	Clinical judgment	Prospective	Single-center	ICU	Plasma	ELISA	60/30	43	7	17	23
Hassuna et al. [38]	2021	Egypt	Children	Clinical judgment	Prospective	Single-center	ICU	Plasma	ELISA	58/72	39	12	19	60
Khera et al. [39]	2022	India	Children	Sepsis-3	Prospective	Single-center	ICU	Plasma	CLEIA	36/18	28	8	8	10
Lee et al. [40]	2022	Korea	Adult	Sepsis-3	Prospective	Single-center	Non-ICU	Plasma	CLEIA	278/142	195	16	83	126
Jeong and Kim [41]	2022	Korea	Adult	Sepsis-3	Prospective	Single-center	ICU	Plasma	CLEIA	129/169	90	48	39	121
Yonaha et al. [42]	2021	Japan	Adult	Sepsis-3	Prospective	Single-center	ICU	Plasma	CLEIA	42/56	37	28	5	28
Klouche et al. [43]	2016	France	Adult	Sepsis-2	Prospective	Multi-center	ICU	Whole blood	CLEIA	100/44	82	13	18	31
Amer et al. [44]	2016	Egypt	Adult	Sepsis-2	Prospective	Single-center	ICU	Whole blood	CLEIA	28/25	23	5	5	20
Enguix-Armada et al. [45]	2016	Spain	Adult	Positive blood culture	Prospective	Single-center	ICU	Plasma	CLEIA	246/142	202	6	44	136
Sato et al. [46]	2015	Japan	Adult	Positive blood culture	Prospective	Single-center	ICU	Whole blood	CLEIA	32/20	23	0	9	20
Venugopalan et al. [47]	2019	India	Adult	Positive blood culture	Prospective	Single-center	ICU	Whole blood	ELISA	26/22	12	0	14	22
Carpio et al. [48]	2015	Peru	Adult	Positive blood culture	Prospective	Single-center	Non-ICU	Plasma	CLEIA	123/123	80	0	43	123
Wang et al. [49]	2020	China	Adult	Sepsis-3	Prospective	Single-center	ICU	Plasma	CLEIA	44/54	36	6	8	48
Roy et al. [50]	2023	India	Adult	Sepsis-3	Prospective	Single-center	ICU	Plasma	ELISA	52/30	41	14	11	16
Hashem et al. [51]	2020	Egypt	Neonate	Positive blood culture	Prospective	Multi-center	ICU	Plasma	CLEIA	133/102	110	5	23	97
Godnic et al. [52]	2015	Slovenia	Adult	Sepsis-2	Retrospective	Single-center	ICU	Plasma	CLEIA	40/7	34	3	6	4
Hincu et al. [53]	2025	Romania	Neonate	Positive blood culture	Prospective	Single-center	ICU	Plasma	ELISA	68/54	46	4	22	50
Piccioni et al. [54]	2025	Romania	Adult	Sepsis-3	Prospective	Single-center	Non-ICU	Plasma	CLEIA	130/86	111	75	19	11
Shakoor et al. [55]	2023	Pakistan	Children	Positive blood culture	Prospective	Single-center	ICU	Plasma	ELISA	22/35	16	10	6	25
Imai et al. [56]	2019	Japan	Adult	Positive blood culture	Prospective	Single-center	Non-ICU	Plasma	CLEIA	46/30	43	18	3	12
Tanir et al. [57]	2018	Türkiye	Children	Positive blood culture	Prospective	Single-center	ICU	Whole blood	ELISA	58/80	58	5	0	75
Wu et al. [58]	2023	China	Adult	Sepsis-3	Prospective	Single-center	ICU	Plasma	CLEIA	164/105	120	26	44	79
Yamamoto et al. [59]	2019	Japan	Adult	Sepsis-3	Prospective	Single-center	ICU	Plasma	CLEIA	62/29	58	4	4	25
Nakamura et al. [60]	2019	Japan	Adult	Sepsis-3	Retrospective	Single-center	ICU	Plasma	CLEIA	146/660	118	112	28	548
Jereb et al. [61]	2019	Slovenia	Adult	Sepsis-3	Prospective	Single-center	ICU	Plasma	CLEIA	54/26	52	4	2	22

Abbreviations: ICU: Intensive care unit; ELISA: Enzyme-linked immunosorbent assay; CLEIA: Chemiluminescent enzyme immunoassay; TP: True positive; FP: False positive; FN: False negative; TN: True negative.

### Quality assessment

The QUADAS-2 assessment results are illustrated in Figure S1. Seventeen studies were classified as having an “unclear” risk of bias in the domains of Patient Selection, Index Test, and Flow and Timing, primarily due to inadequate reporting of diagnostic criteria or clinical context. Additionally, six studies (12.8% of the total) diagnosed sepsis based solely on clinical judgment, lacking a clearly defined standard. Nonetheless, all included studies accurately identified the target condition and independently interpreted index test results without reference to the standard. Overall, the studies exhibited acceptable quality, achieving favorable ratings for both risk of bias and applicability concerns.

### Pooled diagnostic performance metrics

**Figure 2.**
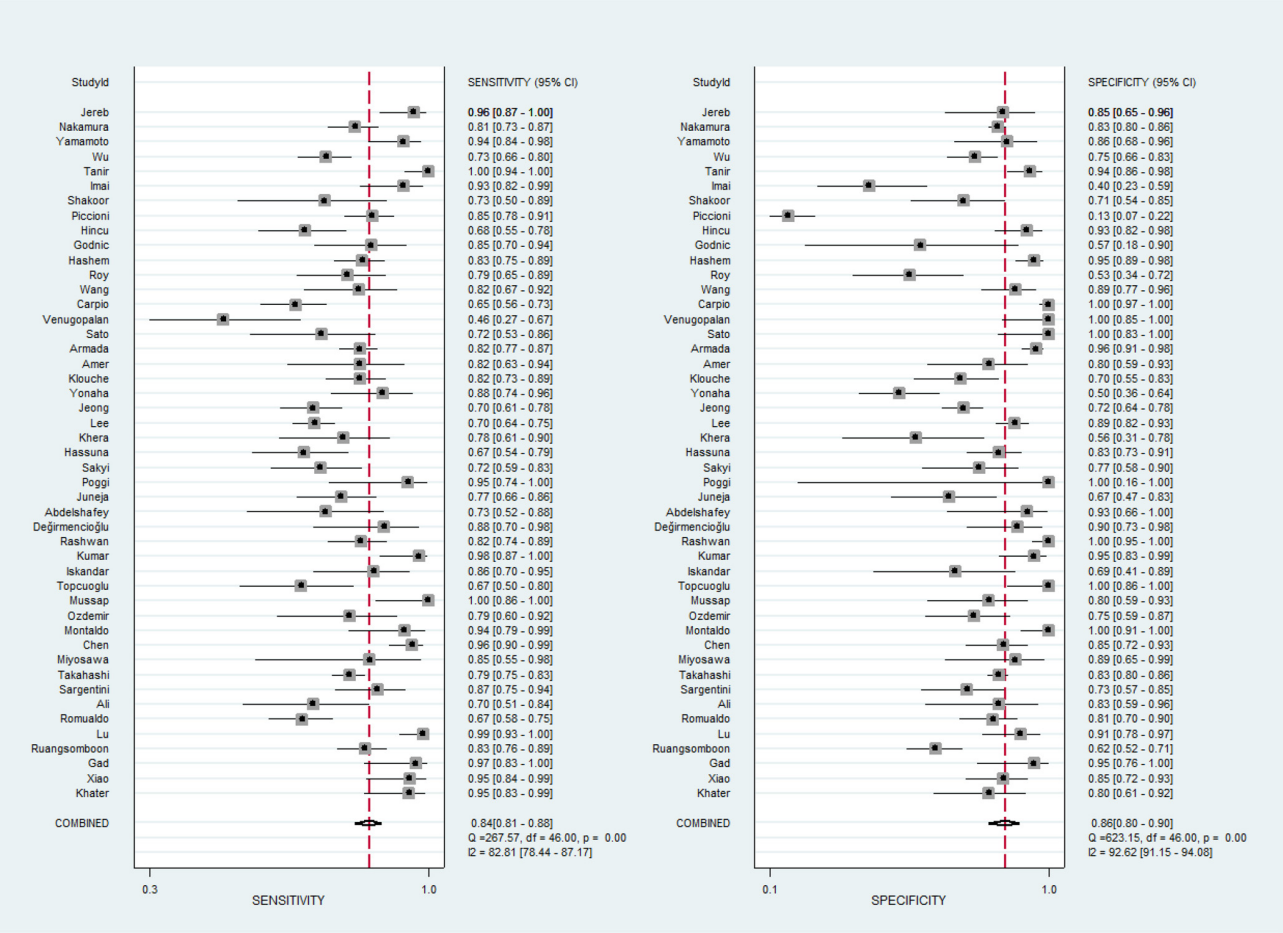
**Pooled sensitivity and specificity of presepsin in the diagnosis of sepsis.** Forest plots showing sensitivity (left) and specificity (right) of presepsin across 47 studies, with pooled estimates calculated using a random-effects model. The analysis demonstrated a sensitivity of 0.84 (95% CI: 0.81–0.88) and specificity of 0.86 (95% CI: 0.80–0.90). Abbreviation: CI: Confidence interval.

**Figure 3.**
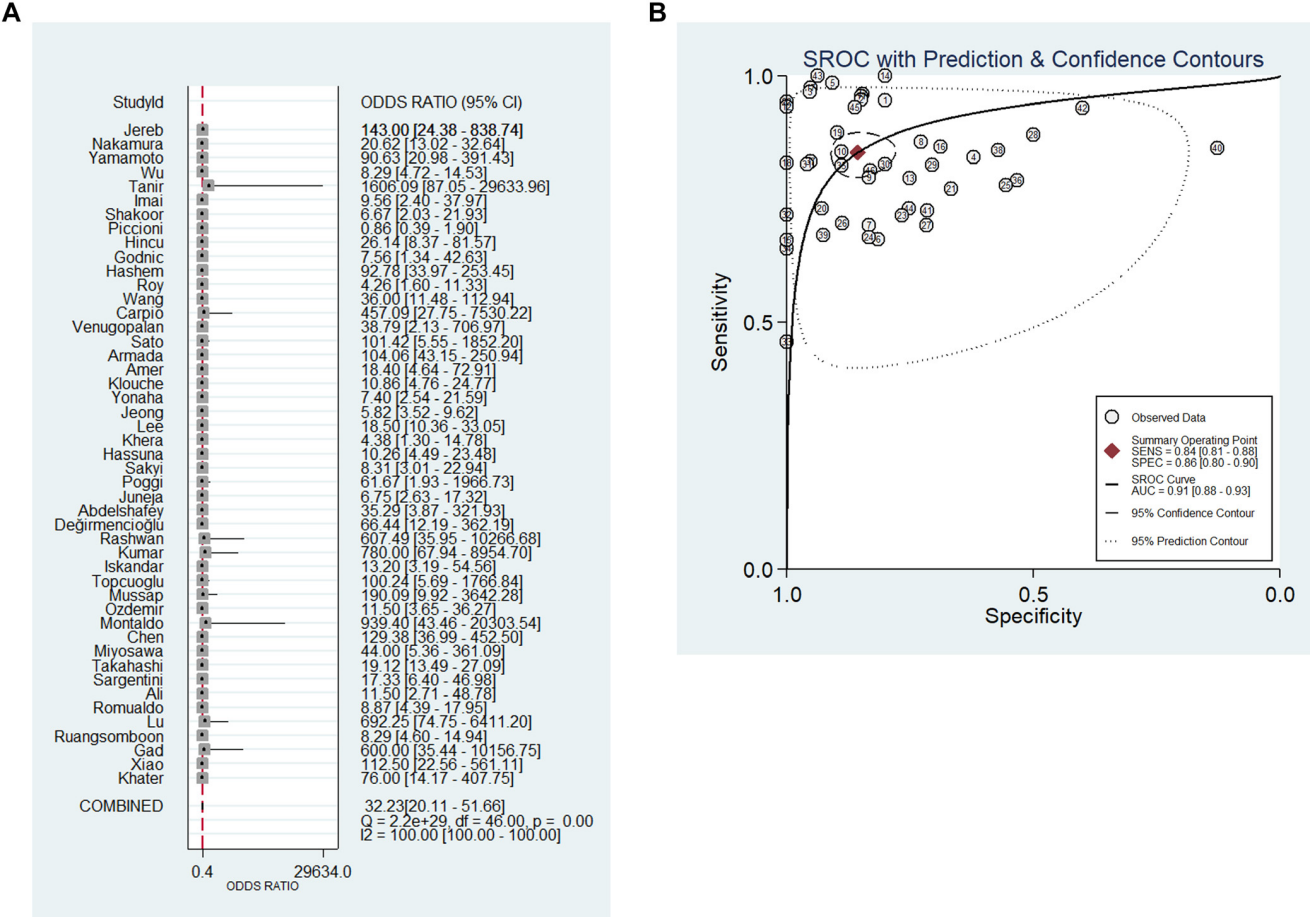
**Pooled diagnostic accuracy of presepsin for sepsis.** (A) Forest plot of DOR with 95% confidence intervals across 47 included studies; (B) SROC curve with 95% confidence and prediction contours. The pooled analysis showed a DOR of 32.23 (95% CI: 20.11–51.66) and an AUC of 0.91 (95% CI: 0.88–0.93), confirming high diagnostic accuracy of presepsin for sepsis. Abbreviations: DOR: Diagnostic odds ratio; SROC: Summary receiver operating characteristic; AUC: Area under the curve; CI: Confidence interval.

The meta-analysis of 47 studies produced the following pooled estimates (95% CI): sensitivity 0.84 (95% CI: 0.81–0.88), specificity 0.86 (95% CI: 0.80–0.90), DOR 32.23 (95% CI: 20.11–51.66), and AUC 0.91 (95% CI: 0.88–0.93) (see Figures 2 and 3). The PLR was 5.86 (95% CI: 4.14–8.29), while the NLR was 0.18 (95% CI: 0.14–0.23) (see Figure S2). Notably, significant heterogeneity was observed across all metrics: sensitivity (*I*^2^ ═ 82.81%), specificity (*I*^2^ ═ 92.62%), PLR (*I*^2^ ═ 95.58%), NLR (*I*^2^ ═ 83.36%), and DOR (*I*^2^ ═ 100%).

### Assessment of heterogeneity

**Figure 4.**
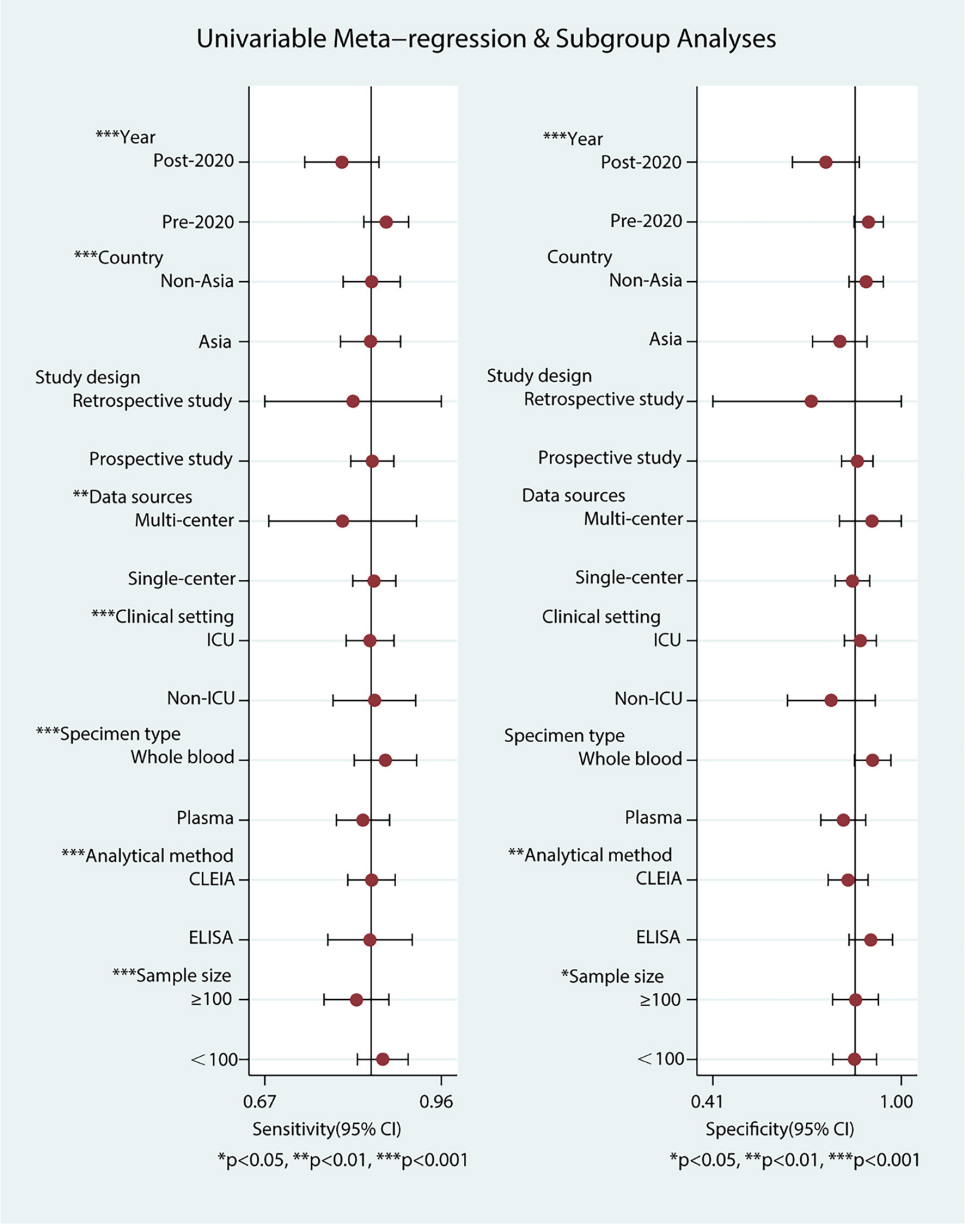
**Univariable meta-regression and subgroup analyses of presepsin diagnostic accuracy.** Forest plots of study-level covariates and their impact on pooled sensitivity (left) and specificity (right). Significant sources of heterogeneity included year, country, specimen type, clinical setting, analytical method, and sample size. Abbreviations: ICU: Intensive care unit; CLEIA: Chemiluminescent enzyme immunoassay; ELISA: Enzyme-linked immunosorbent assay; CI: Confidence interval.

The Spearman correlation coefficient was 0.043 (*P* ═ 0.775), indicating no threshold effect. Meta-regression analysis identified several primary sources of heterogeneity in pooled diagnostic accuracy, including Year (*I*^2^ ═ 84%), Country (*I*^2^ ═ 28%), Specimen type (*I*^2^ ═ 61%), Population (*I*^2^ ═ 87%; *I*^2^ ═ 61%), and Diagnostic criteria (*I*^2^ ═ 50%; *I*^2^ ═ 86%; *I*^2^ ═ 37%; *I*^2^ ═ 72%) (refer to Figure 4, Figure S3 and S4, and Tables S1–S3). Specifically, heterogeneity in sensitivity was significantly influenced by Year (Pre-2020 vs Post-2020), Country (Non-Asia vs Asia), Specimen type (Whole blood vs Plasma), Population (Neonates vs Adults, Children vs Adults), and Diagnostic criteria (Positive blood culture vs Sepsis-2, Positive blood culture vs Sepsis-3, Sepsis-2 vs Sepsis-3, Sepsis-3 vs Clinical judgment). Additionally, Year (Pre-2020 vs Post-2020) and Diagnostic criteria (Positive blood culture vs Sepsis-3, Sepsis-3 vs Clinical judgment) were key determinants of specificity heterogeneity. Although residual within-group heterogeneity persisted for both sensitivity and specificity, meta-regression analysis indicated an *I*^2^ of 0%, suggesting a minimal impact on overall heterogeneity.

To further investigate heterogeneity, we conducted subgroup analyses of diagnostic performance metrics, as outlined in Table 2. Pre-2020 studies exhibited superior diagnostic performance compared to Post-2020 studies (AUC 0.95 vs 0.83). While country contributed to heterogeneity, sensitivity remained consistent across Non-Asian and Asian cohorts (0.84), with divergence primarily evident in PLR (8.25 vs 4.20) and DOR (47.76 vs 21.52). Whole blood and plasma samples demonstrated comparable sensitivities (0.88 vs 0.83); however, Whole blood showed significantly higher PLR (8.81 vs 4.60), DOR (66.23 vs 21.70), and AUC (0.95 vs 0.88). Presepsin exhibited exceptional diagnostic accuracy in Neonates (sensitivity 0.90, specificity 0.92, PLR 10.94, NLR 0.11, DOR 101.99, AUC 0.96), while maintaining robust performance in Children (AUC 0.88) and Adults (AUC 0.87). Significant heterogeneity was noted across various diagnostic criteria. Positive blood culture achieved optimal diagnostic performance (sensitivity 0.85, specificity 0.94, PLR 15.46, NLR 0.16, DOR 97.5, AUC 0.94), followed by clinical judgment (AUC 0.92), Sepsis-2 (AUC 0.89), and Sepsis-3 (AUC 0.83).

**Table 2:** Pooled diagnostic estimates across subgroups

**Subgroup**	**Number of studies**	**Sensitivity (95% CI)**	**Specificity (95% CI)**	**PLR (95% CI)**	**NLR (95% CI)**	**DOR (95% CI)**	**AUC (95% CI)**
*Year*							
Post-2020	18	0.78 (0.74–0.82)	0.76 (0.65–0.85)	3.32 (2.2–5.01)	0.28 (0.23–0.34)	11.75 (6.8–20.31)	0.83 (0.79–0.86)
Pre-2020	29	0.88 (0.83–0.91)	0.9 (0.84–0.94)	8.74 (5.43–14.05)	0.14 (0.10–0.19)	63.79 (34.94–116.48)	0.95 (0.92–0.96)
*Country*							
Non-Asia	25	0.84 (0.79–0.89)	0.9 (0.82–0.95)	8.25 (4.51–15.08)	0.17 (0.13–0.24)	47.76 (22.67–100.6)	0.92 (0.9–0.94)
Asia	22	0.84 (0.79–0.89)	0.8 (0.72–0.86)	4.2 (3–5.87)	0.2 (0.14–0.27)	21.52 (12.22–37.9)	0.89 (0.86–0.92)
*Population*							
Neonates	16	0.90 (0.84–0.94)	0.92 (0.86–0.95)	10.94 (6.38–18.76)	0.11 (0.07–0.17)	101.99 (50.61–205.54)	0.96 (0.94–0.98)
Children	5	0.84 (0.58–0.95)	0.81 (0.67–0.89)	4.31 (2.09–8.88)	0.20 (0.06–0.66)	21.16 (3.37–132.99)	0.88 (0.85–0.9)
Adults	26	0.81 (0.76–0.85)	0.82 (0.72–0.89)	4.41 (2.81–6.91)	0.23 (0.19–0.29)	18.84 (11.04–32.16)	0.87 (0.84–0.9)
*Diagnostic criteria*							
Positive blood culture	19	−0.85 (0.78–0.9)	−0.94 (0.87–0.98)	−15.46 (6.38–37.46)	−0.16 (0.11–0.23)	−97.5 (39.78–238.97)	−0.94 (0.92–0.96)
Sepsis-2	6	0.87 (0.78–0.92)	0.8 (0.72–0.87)	4.43 (2.96–6.63)	0.16 (0.09–0.29)	26.9 (11.04–65.56)	0.89 (0.86–0.92)
Sepsis-3	16	0.8 (0.75–0.84)	0.73 (0.62–0.82)	2.97 (2.04–4.33)	0.28 (0.22–0.35)	10.7 (6.25–18.31)	0.83 (0.8–0.86)
Clinical judgment	9	0.92 (0.79–0.97)	0.87 (0.81–0.91)	7.01 (4.46–11.02)	0.09 (0.03–0.27)	77.16 (18.4–323.61)	0.92 (0.89–0.94)
*Study design*							
Prospective study	44	0.85 (0.81–0.88)	0.86 (0.8–0.91)	6.25 (4.3–9.09)	0.18 (0.14–0.23)	35.58 (21.46–58.98)	0.92 (0.89–0.94)
*Data sources*							
Multi-center	5	0.79 (0.75–0.83)	0.9 (0.76–0.97)	8.33 (3.09–22.44)	0.23 (0.19–0.27)	36.22 (12.91–101.61)	0.83 (0.79–0.86)
Single-center	42	0.85 (0.81–0.89)	0.85 (0.78–0.9)	5.63 (3.9–8.15)	0.17 (0.13–0.23)	32.36 (19.18–54.59)	0.91 (0.89–0.94)
*Clinical setting*							
ICU	37	0.84 (0.8–0.88)	0.87 (0.82–0.9)	6.3 (4.49–8.82)	0.18 (0.14–0.24)	34.49 (20.96–56.75)	0.92 (0.89–0.94)
Non-ICU	10	0.85 (0.76–0.91)	0.79 (0.55–0.92)	4.11 (1.70–9.95)	0.19 (0.11–0.31)	18.95 (4.87–73.7)	0.89 (0.86–0.92)
*Specimen type*							
Whole blood	17	0.88 (0.8–0.93)	0.9 (0.83–0.94)	8.81 (5.04–15.4)	0.13 (0.08–0.23)	66.23 (28.14–155.87)	0.95 (0.93–0.97)
Plasma	30	0.83 (0.78–0.86)	0.82 (0.73–0.88)	4.6 (3.06–6.92)	0.21 (0.17–0.27)	21.7 (12.68–37.11)	0.88 (0.85–0.91)
*Analytical method*							
CLEIA	35	0.84 (0.8–0.87)	0.83 (0.76–0.89)	5.08 (3.46–7.45)	0.19 (0.15–0.24)	26.51 (16.12–43.6)	0.9 (0.87–0.92)
ELISA	12	0.86 (0.74–0.93)	0.9 (0.81–0.95)	8.72 (4.35–17.49)	0.16 (0.08–0.31)	55.99 (17.64–177.69)	0.92 (0.92–0.96)
*Sample size*							
≥ 100	20	0.82 (0.76–0.87)	0.86 (0.76–0.92)	5.87 (3.33–10.34)	0.21 (0.16–0.28)	27.86 (13.36–58.11)	0.89 (0.86–0.92)
< 100	27	0.86 (0.81–0.9)	0.85 (0.77–0.9)	5.71 (3.74–8.71)	0.16 (0.11–0.22)	35.83 (19.69–65.21)	0.92 (0.9–0.94)

Abbreviations: PLR: Positive likelihood ratio; NLR: Negative likelihood ratio; DOR: Diagnostic odds ratio; AUC: Area under the curve; ICU: Intensive care unit; CLEIA: Chemiluminescent enzyme immunoassay; ELISA: Enzyme-linked immunosorbent assay.

### Robustness and publication bias

**Figure 5.**
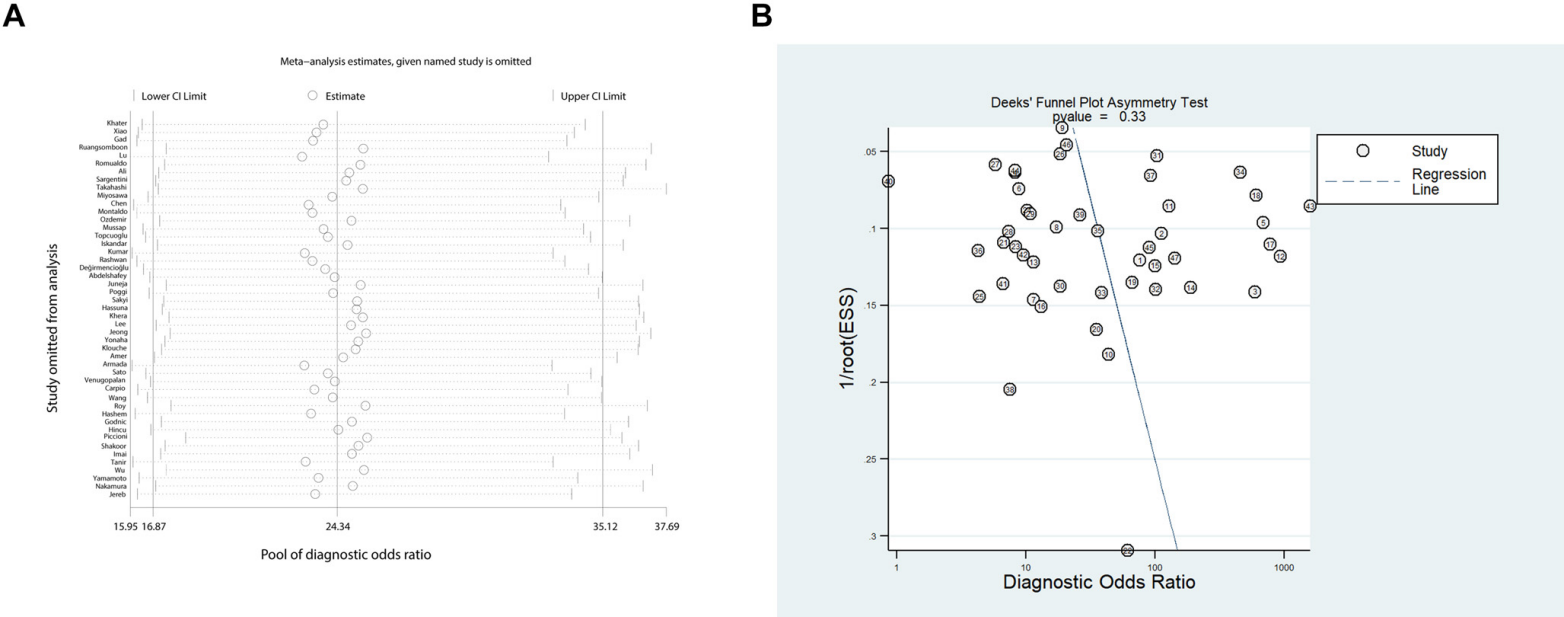
**Sensitivity analysis and publication bias for presepsin diagnostic performance.** (A) Leave-one-out influence analysis for the DOR, where each circle represents the pooled estimate with its 95% CI when the corresponding study is excluded; (B) Deeks’ funnel plot used to evaluate potential small-study effects and publication bias, with circles representing individual studies and the regression line assessing symmetry. Abbreviations: DOR: Diagnostic odds ratio; CI: Confidence interval; ESS: Effective sample size.

A sensitivity analysis of 47 studies, including 41 studies with a clearly defined standard, confirmed the robustness of the meta-analytic findings, indicating that no single study exerted undue influence on the pooled estimates (Figure 5A, Figure S5). Additionally, Deeks’ funnel plot asymmetry test indicated no significant publication bias (*P* ═ 0.33, Figure 5B).

## Discussion

This meta-analysis demonstrates that, despite variability in factors such as year, country, specimen type, population, and diagnostic criteria, presepsin exhibits significant clinical utility and outstanding overall diagnostic performance. The pooled sensitivity is 0.84 (95% CI: 0.81–0.88), specificity is 0.86 (95% CI: 0.80–0.90), DOR is 32.23 (95% CI: 20.11–51.66), and AUC is 0.91 (95% CI: 0.88–0.93).

Presepsin, a crucial mediator of innate immune activation, is cleaved and released into circulation upon the binding of pathogen-associated molecular patterns to the CD14 receptor. Its levels increase significantly within three hours post-infection and demonstrate high specificity for bacterial sepsis [52, 62, 63]. Presepsin rises earlier and more rapidly than established sepsis biomarkers during systemic inflammation [64].

In contrast to the study by Liang and Su [65], which evaluated the mortality rate of sepsis through the systemic inflammation index, our research expands the meta-analysis of sepsis biomarkers.

Year, country, specimen type, population, and diagnostic criteria were identified as significant sources of heterogeneity. The heterogeneity associated with the year (*I*^2^ ═ 84%) indicated superior diagnostic accuracy in pre-2020 studies (AUC 0.95) compared to post-2020 studies (AUC 0.83). This difference is attributable to evolving diagnostic standards, particularly the implementation of more stringent sepsis definitions under Sepsis-3. Despite exhibiting identical sensitivity (0.84), non-Asian cohorts demonstrated higher PLR (8.25) and DOR (47.76). Whole blood specimens consistently outperformed plasma in diagnostic parameters, indicating their high quality for enhanced diagnostic performance. In subgroup analyses based on diagnostic criteria, the AUCs for positive blood culture, Sepsis-2, Sepsis-3, and clinical judgment were 0.94, 0.89, 0.83, and 0.92, respectively. Notably, positive blood culture serves as an earlier and more permissive diagnostic standard, while clinical judgment emphasizes experience-based diagnostic approaches. In contrast to the more stringent and clinically aligned Sepsis-3 criteria, the diagnostic accuracy of positive blood culture and clinical judgment requires cautious interpretation. Nevertheless, presepsin demonstrated robust performance across all subgroups of diagnostic criteria, maintaining a minimum AUC of 0.83, thereby affirming its utility as a promising biomarker.

Presepsin exhibited optimal diagnostic performance in neonates, with a sensitivity of 0.90, specificity of 0.92, and AUC of 0.96. This efficacy can be attributed to the innate immune status of neonates, which facilitates robust CD14 pathway activation, an underdeveloped blood-brain barrier that allows for rapid release during central nervous system infections, and stable renal clearance that minimizes false elevations [9, 66, 67]. Our findings in neonates (AUC 0.96) align with those of Poggi et al. [68], who reported a meta-analysis of neonates with an AUC of 0.96, sensitivity of 0.93, and specificity of 0.91.

Presepsin also demonstrated substantial diagnostic accuracy in children (AUC 0.88) and adults (AUC 0.87), with our adult specificity surpassing that of adult meta-analyses published in 2015 (0.78) [69]. This improvement may reflect optimized diagnostic criteria or enhanced assay sensitivity. However, the AUC for children (0.88) in our study was lower than that reported in the children’s meta-analysis (AUC 0.93) [70], potentially due to the limited number of pediatric studies (*n* ═ 5), which may have reduced diagnostic precision. Furthermore, developmental dynamics in CD14 expression and immunocyte reactivity could attenuate presepsin kinetics [71], while frequent viral coinfections may diminish the specificity for bacterial sepsis due to reduced CD14 pathway engagement by viral pathogens [72].

Given immunological fluctuations, limited data, and frequent viral confounders [71], [72], presepsin is a primary biomarker for diagnosing sepsis in neonates and adults. For pediatric patients, we recommend using a multimarker panel that combines presepsin, PCT, and high-sensitivity CRP to facilitate early-stage sepsis detection [37]. This study quantified the differential diagnostic accuracy across populations, revealing an AUC of 0.96 for neonates, 0.88 for children, and 0.87 for adults.

However, several limitations must be acknowledged, including inadequate statistical power in the pediatric subgroup (*n* ═ 5 studies), restricted generalizability due to predominance of ICU-based sampling, and potential diagnostic performance bias from non-standardized specimen processing, which may lead to CD14 degradation during coagulation. To address these limitations, large-scale, prospective, multicenter studies are urgently needed. These studies should encompass diverse geographic regions and healthcare environments, including community hospitals and emergency departments, with a focus on underrepresented populations, particularly children, to validate diagnostic accuracy.

Furthermore, future research should aim to clarify the biological mechanisms that influence the diagnostic performance of presepsin across different populations—neonates, children, and adults—particularly during the dynamic immune responses observed in children. Finally, it is essential to standardize sample detection protocols, including direct comparisons of plasma vs whole blood performance under controlled conditions for collection, processing, and storage. Notably, none of the studies reported etiology data for sepsis cases, making it impossible to analyze data related to specific causative pathogens or etiological categories. Future research that establishes subgroups based on sepsis etiology could provide valuable insights into the biological mechanisms underlying presepsin and enhance its diagnostic efficacy.

## Conclusion

This meta-analysis establishes presepsin as a highly promising sepsis biomarker. Its application in pediatric patients requires validation through large prospective studies.

## Supplemental data

Supplemental data are available at the following link: https://www.bjbms.org/ojs/index.php/bjbms/article/view/12909/3988.

## Data Availability

All data generated during this study are included in the manuscript.
